# Factors associated with choice of antenatal, delivery and postnatal services between HIV positive and HIV negative women in Zambia

**DOI:** 10.1186/s12884-019-2272-0

**Published:** 2019-04-15

**Authors:** Choolwe Muzyamba, Wim Groot, Milena Pavlova, Sonila M. Tomini

**Affiliations:** 1 Maastricht Graduate School of Governance, UNU-Merit, Maastricht University, Maastricht, Netherlands; 2 A9 Marshlands Village Box 32379, Lusaka, Zambia; 3 Department of Health Services Research, CAPHRI; Maastricht University Medical Center; Faculty of Health, Medicine and Life Sciences; Maastricht University, Maastricht, Netherlands; 4 Top Institute for Evidence-Based Education Research (TIER), Maastricht University, Maastricht, Netherlands; 5 Department of Economics, University of Liege, Liege, Belgium

**Keywords:** Skilled attendants, Traditional birth attendants, HIV, Maternal health

## Abstract

**Background:**

Previous research has shown that developing countries account for the majority of maternal deaths around the world. Relatively high maternal mortality in developing countries has been linked to high HIV prevalence rates in these countries. Several studies have shown that women living with HIV are more vulnerable and are thus more likely to die during maternity than those who are not. Although there has been increased focus on this subject in contemporary research, the relationship between HIV status and maternal-care-utilization is not very well understood. It is not clear whether factors associated with professional maternal care utilization during antenatal, delivery and postnatal periods are similar for HIV positive and HIV negative women. It is also not known whether being HIV positive has an impact on the choice of care (professional care or traditional birth attendants). Thus the aim of this study is to investigate the differences in factors affecting choice of care during antenatal, delivery and postnatal periods between HIV positive and HIV negative women. We also investigate the effect of HIV positive status on choice of care.

**Methods:**

By using the 2013–2014 Zambia Demographic Health Survey Data (ZDHS), we performed two different quantitative analyses. a) Regression analysis: to identify and compare factors associated with the likelihood of utilizing professional care during antenatal, at birth and postnatal periods between HIV positive and HIV negative women. b) Propensity score matching: to investigate the effect of being HIV positive on the choice of care (Professional care or TBAs).

**Results:**

Our results show that reasons for choosing professional care during antenatal, at birth, and postnatal periods are the same for both HIV positive and HIV negative women. Further, we also showed that although the probability of utilizing professional care is slightly higher for HIV positive women, the difference is negligible.

**Conclusion:**

We demonstrated that in Zambia, utilization of professional care among HIV positive women is not particularly high. We also demonstrate that although institutional care is desirable and an ideal solution for HIV positive women, insisting on institutional care when the health facilities lack adequate trained personnel, drugs, and equipment is counterproductive.

## Background

Developing countries account for over 99% of all maternal deaths around the world [1]. The situation is even worse in high HIV-prevalent countries like Zambia [2, 3]. Sebitloane et al. 2009 showed that high maternal deaths among HIV positive women are as a result of both obstetric causes (such as puerperal sepsis, obstetric hemorrhage etc.) and non-obstetric causes (such as tuberculosis, tetanus, malaria etc.). In an effort to reduce the risk of maternal mortality, the World Health Organization (WHO) recommends consistent utilization of skilled maternal care services by all HIV positive women which simultaneously promotes Prevention-of-Mother-to-Child-Transmission of HIV. Professional care in maternal health usually involves early and focused antenatal care, delivering in a health facility under skilled supervision and use of skilled postnatal care [4]. Maternal health experts suggest that in order to reduce maternal mortality in HIV positive women, professional care should be present across all three stages of care (antenatal, birth and postnatal stage) [5]. There seems to be consensus among public health professionals that utilization of skilled maternal care services from the onset of pregnancy to postnatal period reduces negative maternal health outcomes in HIV positive women. This results from the consistent uptake of antiretroviral treatment (ART) and giving birth by caesarian section. These two actions are expected to collectively promote PMTCT and also improve the health of the mother and the child [6, 7]. Despite all its benefits, professional care usage has remained unsatisfactory in most parts of Sub-Saharan Africa (SSA). HIV positive women continue to utilize services of Traditional Birth Attendants (TBAs) instead of professional care [6, 7]. This means that the ‘game-changing potential’ of antiretroviral treatment aimed at radically reducing HIV-related maternal deaths is not being realized [8–10].

Further, while the current body of research has established a link between high maternal mortality and HIV status, there still remains a gap in understanding the relationship between HIV status and maternal health utilization [11, 12]. It is not yet clear whether factors associated with professional maternal care utilization during antenatal, delivery and postnatal periods are similar for both HIV positive and HIV negative women. It is also not yet known whether being HIV positive has an impact on choice of care (professional care or TBAs). Given the risks associated with HIV positive status, it is expected that the propensity to seek professional care is higher among HIV positive women [2, 9]. However, the extent to which this is evident in Zambia is still unclear [9]. Establishing this link could be the first step towards the formulation of effective policy aimed at promoting maternal health among the most vulnerable women [13]. Thus the aim of this study is a) to investigate and compare factors associated with the choice of care during antenatal, delivery and postnatal periods between HIV positive and HIV negative women. b) to investigate the effect of HIV positive status on the utilization of professional maternal care during antenatal, delivery and postnatal periods.

## Methods

This study made use of the publicly available and nationally representative survey known as the 2013–2014 Zambia Demographic Health Survey (ZDHS). This is a survey that was conducted by a consortium of institutions including the Zambian ministry of health, the University of Zambia and the Virology Laboratory at the University Teaching Hospital of Zambia ([2], p. 14). The ZDHS contains social demographic data obtained from women aged 15–49 years. The sample in the ZDHS 2013–2014 is a two-stage stratified cluster sample design. In the first stage, 722 Enumeration Areas (EAs) (305 in urban areas and 417 in rural areas) were selected from all the 10 provinces of Zambia. Stratification was done by dividing each of the 10 provinces into urban and rural areas resulting in 20 sampling strata. In the second stage, a complete list of households was used as a sampling frame upon which 25 households were selected for enumeration in each EA [14]. It was at this stage that a representative sample of 18,052 households was finally selected.

Regarding HIV results; the HIV data were obtained by collecting blood samples during interviews from consenting participants (83.6% of the total sample) and later testing the blood samples for HIV and then linking the sample results to the unique IDs of participants. Specifically, after fulfilling the country’s ethical requirements, the DHS team collected blood spots on filter paper from a finger prick and transported them to a laboratory for testing. If the respondent did not consent to testing, their HIV status was left unstated in the data and treated as missing ([8], p. 14). The ZDHS does not make the HIV data publicly available, as such, these data were only availed to us after obtaining ethical clearance from the Ethics board of Zambia and the ministry of health in Zambia. By using unique IDs, we then linked the HIV data to the rest of the dataset.

The next step was to select a sub-sample of women who had given birth at least once in the last 5 years preceding the survey and had a determined HIV status. We did not include women who did not take part in the HIV test or had undetermined HIV status. Our final sample thus consisted of 12, 225 women. These data enabled us to investigate the difference in maternal health care utilization (skilled delivery attendants or trained TBAs) between HIV-positive and HIV-negative women in Zambia. A summary of the characteristics of the data is provided in Table 1.

**Table 1:** A summary of characteristics (differences and similarities) between HIV positive and HIV negative populations. (Which we summarized in our previous study

Variable (*N* = 12, 225)		Frequency		Valid Percentage (%)		(t) and (χ^2^)
		HIV +	HIV-	HIV +	HIV-	
Type of residence	Urban	1014	4752	48.66	47.85	103.151***
	Rural	1098	5359	51.34	52.15	17.117***
	Total	2113	10, 112			
Religion	catholic	369	1792	17.47	17.72	17.011***
	protestant	1722	8209	81.50	81.19	13.060***
	Muslim	10	50	0.45	0.49	1.251**
	other	12	62	0.58	0.60	0.015***
	Total	2113	10,112			
Education	no education	165	1187	7.8	11.74	23.110***
	primary	951	5747	45.54	56.83	9.463***
	secondary	845	2811	40.50	27.80	16.717***
	higher	128	404	6.11	4.01	76.701***
	Total	2113	10,112			
Wealth index	poorest	374	1742	17.72	17.23	121.123***
	poorer	364	1866	17.23	18.45	10.104***
	middle	464	2154	21.50	21.24	12.330***
	richer	467	2053	21.37	20.91	8.145***
	richest	468	2225	22.17	22.18	109.204***
	Total	2113	10,112			
Age	Age 15–25	972	4045	46	40.61	111.020**
	Age 26–35	592	3235	28	32.26	20.511***
	Age 36–45	549	2732	26	27.14	0.500**
	Total	2113	10,112			
Health insurance	No health insurance	2066	9808	97.82	97.40	91.166**
	With health insurance	46	303	2.18	2.60	76.661***
	Total	2113	10,112			
Availability of drugs in facility	Big problem	586	4142	27.74	40.96	10.221***
	Not a big problem	1527	5970	72.26	59.04	12.770***
	Total	2113	10,112			
Distance to facilitates	Big problem	795	4348	37.64	43.95	2.091***
	Not a big problem	1318	5764	63.26	59.05	12.413**
	Total	2113	10,112			
Attitude of health professionals	Big problem	549	3369	26.40	33.32	8.543***
	Not a big problem	1564	6743	73.60	66.68	4.500***
	total	2113	10,112			
Choice of care at Antenatal	TBA	486	2375	23.00	23.49	0.828***
	Health professional	1527	7737	77.00	76.51	2.798***
	total	2113	10,112			
Choice of care at birth	TBA	448	2124	21.10	21.00	16.223***
	Health professional	1665	7989	78.90	79.00	18.123***
	Total	2113	10,112			
Choice of care at Postnatal	TBA	507	2395	24.01	23.68	13.352***
	Health professional	1606	7718	75.09	76.32	19.113***
	total	2113	10,112			

***p < 0.01, **p < 0.05, *p < 0.10. Reported *p*-values are based on ttests of means for continuous variables and chi-squares for proportions/categorical variables

For the selection of explanatory variables, we relied on previous studies on maternal health utilization [2, 9, 10], and also made use of the Andersen’s behavioral model of health which holds that usage of any given health service is based on three dynamics: predisposing factors (such as socio-demographic factors), enabling factors (such as wealth, access to health insurance) and healthcare needs (chronic illnesses, functional disability etc.) [15]. We therefore included the following predisposing factors in our study: age, religion, area of residence, level of education, distance to health facility, attitude of health workers and availability of drugs in facilities. The enabling factors were wealth and health insurance. While having given birth at least once in the last 5 years is seen as a proxy for the ‘need’ factor.

The ZDHS included a section on maternal health service utilization for the most recent birth. In this section, questions were asked on who attended to the women during antenatal, birth and postnatal periods. The options in general included: Health professional and TBAs. Therefore, we separately use this dummy variable (health professional or TBA) for all the three stages of maternal health utilization (antenatal, birth, and postnatal stage).

We undertook two different analyses: *Probit analysis*: to identify the factors that influenced the likelihood of utilizing professional care during antenatal, at birth and postnatal (for both HIV positive and HIV negative women). On the basis of this probit analysis, we further generated marginal effects to ascertain the probabilities of utilization of professional care during antenatal, at birth and postnatal. *Propensity Score Matching:* to investigate the effect of being HIV positive on choice of maternal health services between professional care and TBA during antenatal, at birth and postnatal. For this, we calculate the Average Treatment Effect on the Treated (ATT).

More specifically, Propensity Score Matching (PSM) is used to allow us to statistically formulate a control group (HIV negative women) by matching the observed characteristics of the treated participants (HIV positive women) to the control group. This is based on similar values of the propensity score [16]. Heckman et al. (1998) define PSM as the probability of selection into the treated group, which in this case means the probability of being HIV positive. The variables that we use for matching include age, educational level, area of residence, and wealth. The selection of variables was guided by theory and consensus in extant literature regarding what factors are likely to increases chances of being HIV positive [17–19]. When conducting PSM, it is important to note that the “unbiased inference” in PSM is based on the assumption that outcomes are independent of assignment to the treatment group on the basis of observable characteristics [20]. In order to have valid results, it is important that between the propensity scores of treatment and control groups, there exists an area of “common-support” [21].

We used Stata to estimate the ATT using the Nearest Neighbor Matching technique [22]. This is a technique that matches individuals from control and treatment group with similar propensity scores and then drops all those that are not selected in the match [16].

The reliability of the estimated effect of the HIV positive status on choice of maternal care when using PSM depends on selection of observables [20]. For this purpose, we inspected the propensity score distribution to confirm the existence of common support area and we also checked for the balancing property using psmatch2 [23].

Regarding the robustness of the ATT, we compared our findings resulting from the Nearest Neighbor matching technique with two other matching techniques namely Kernel matching and Stratification matching [24]. Kernel matching makes use of more information which allows it to lower the variance [20]. Stratification matching on the other hand partitions the common support into different strata and then computes the impact of treatment within each of those strata [24]. What goes on specifically within the strata is that the effect of the HIV positive status is established as the mean difference in outcomes (use of skilled birth attendant or TBA) between the control and the treated individuals. An average of the interval impacts which is weighted gives the overall HIV status impact by taking the share of the individuals in each interval as weights [25].

### Hidden bias and sensitivity test

PSM has a weakness of failing to correct for biases resulting from unobservables. Thus to deal with this challenge we use the Altonji et al. test. This test allows us to estimate the sensitivity of selection on the basis of unobserved characteristics [20].

## Results

The probit analysis helped us to identify factors that are associated with the likelihood of utilizing professional care during antenatal, at birth and postnatal periods for both HIV positive and HIV negative women. We calculated marginal effects of the explanatory variables to ascertain the probabilities of utilization of professional care during antenatal, at birth and postnatal periods. For the results, see Tables 2 and 3.

**Table 2:** Probit results at antenatal, birth and postnatal for birth HIV+ and HIV

Professional care = 1 TBA = 0		Antenatal				At Birth				postnatal			
		HIV-		HIV+		HIV-		HIV+		HIV-		HIV+	
Model detail		N. of obs = 10,112 Prob > chi2 = 0.0000 Pseudo R2 = 0.0824		N. of obs = 2113 Prob > chi2 = 0.0000 Pseudo R2 = 0.771		N. of obs = 10,112 Prob > chi2 = 0.0000 Pseudo R2 = 0.0915		N. of obs = 2113 Prob > chi2 = 0.0000 Pseudo R2 = 0.0846		N. of obs = 10,112 Prob > chi2 = 0.0000 Pseudo R2 = 0.0522		N. of obs = 2113 Prob > chi2 = 0.0000 Pseudo R2 = 0.1115	
Independent variables		Coef	SE	Coef	SE	Coef	SE	Coef	SE	Coef	SE	Coef	SE
Possession of insurance		0.66	0.041	−0.043	0.001	−0.26	0.000	0.03	0.008	0.26	0.011	0.32	0.001
Availability of Drugs in health centers		0.66*	0.010	.029*	0.003	−0.04	0.005	0.09	0.018	0.01*	0.106	0.00	0.090
positive attitude of workers in health centers		0.13	0.091	0.01	0.002	0.06	0.013	0.04	0.002	0.38*	0.102	0.05*	0.000
Distance to facility		0.53	0.008	0.11	0.050	0.21	0.003	0.01	0.001	0.19	0.033	0.56	0.077
Wealth^a^	Poorer	0.04	0.022	0.34*	0.067	0.28**	0.121	−0.01	0.012	0.87	0.101	0.33**	0.863
	Middle	0.01	0.010	0.01*	0.044	−0.54*	0.141	0.02	0.000	0.66	0.000	0.21**	0.777
	Richer	.015*	0.018	0.00	0.010	0.60*	0.031	0.05**	0.618	.042*	0.118	0.04*	0.103
	Richest	.02**	0.229	0.16**	0.468	0.31**	0.813	0.07**	0.728	0.02**	0.133	0.10**	0.174
Religion^b^	Protestant	0.86	0.001	0.01	0.002	0.98	0.012	0.01	0.009	0.09	0.003	0.81	0.008
	Muslim	0.38	0.018	0.08	0.071	−0.44	0.000	0.06	0.014	0.51	0.000	−0.29	0.011
	Other	0.01	0.003	−0.01	0.131	0.48	0.012	0.06	0.010	0.01	0.040	−0.04	0.000
Education^c^	Primary	−0.00*	0.038	.02**	0.020	0.60***	0.372	0.02*	0.023	0.02*	0.001	0.45**	0.766
	Secondary	.011*	0.013	0.00	0.026	.014*	0.170	0.01**	0.231	0.01	0.022	0.60*	0.122
	higher	0.02***	0.839	.025**	0.862	0.52*	0.107	0.01**	0.871	0.31**	0.138	0.22**	0.994
Type of residence (0 = Urban, 1 = Rural)		0.02***	0.539	0.41***	0.891	0.80***	0.445	0.15***	0.339	0.80***	0.524	0.40***	0.888
Age		0.03	0.003	0.04	0.000	0.04	0.000	0.04	0.000	−0.01	0.003	0.04	0.000
Constant		0.85***	0.968	1.71***	0.881	0.12***	0.741	1.85***	0.913	1.20***	0.947	2.10***	0.846

***p < 0.01, **p < 0.05, *p < 0.10

^a^reference is poorest

^b^reference is catholic

^c^Reference is no education

**Table 3:** Marginal effects of the probit models. Dependent variable is probability of utilizing professional care. Maximum likelihood probit was used during estimation

Professional care =1,TBA = 0		Antenatal		At Birth		Postnatal	
Dependent Variables		HIV-	HIV+	HIV-	HIV+	HIV-	HIV+
Possession of insurance		0.01	0.00*	0.01	0.00	0.00	0.00
Availability of Drugs at clinic		0.33	0.05	0.03*	0.09	0.01	0.00
positive attitude of staff at clinic		0.00	0.08	0.07	0.43	0.00**	0.03
Availability of Staff t clinic		0.00*	0.03*	0.03	0.08*	0.04	0.00
Distance to facility		0.08	0.24	0.55	0.07	0.11	0.35
Wealth^a^	Poorer	0.02*	0.09	0.08*	0.028*	0.07**	0.01*
	Middle	0.00	0.00**	0.00*	0.01	0.00	0.01**
	Richer	0.01***	0.01	0.01**	0.03**	0.09*	0.04
	Richest	0.03**	0.06***	0.02**	0.01***	0.00*	0.01***
Religion^b^	Protestant	0.08	0.01	0.02	0.08	0.09	0.08
	Muslim	0.02	0.00	0.01	0.09	0.03	−0.02
	Other	0.01	−0.06	0.04	0.03	0.01	−0.02
Education^c^	Primary	0.00*	0.04**	0.00***	0.01*	0.04*	0.02**
	Secondary	0.03*	0.00	0.01*	0.02**	0.00*	0.01*
	higher	0.03***	0.04**	0.01**	0.01**	0.01**	0.07**
Type of residence		0.03***	0.02***	0.08***	0.00***	0.04***	0.06***
Age		0.11	0.01	0.01	.004	0.01	0.03
Sample size		12, 225	12, 225	12, 225	12, 225	12, 225	12, 225

***p < 0.01, **p < 0.05, *p < 0.10

^a^reference is poorest

^b^reference is catholic

^c^Reference is no education

Our results show that reasons for choosing professional care over TBAs were similar for both HIV positive and HIV negative women. For example, in both cases wealth, level of education and area of residence influenced women’s choice of care. Specifically, being richest increased the likelihood of utilizing professional care by 4% among HIV negative women and by 6% among HIV positive women. Attaining higher education increased the probability of utilizing professional antennal care by 3% among HIV negative women and 4% among HIV positive women. Similarly, residing in urban areas increased the probability of utilizing professional care by 3% among HIV negative women and 2% among HIV positive women.

At birth, attaining the highest level of wealth increased the probability of utilizing professional care by 2% among HIV negative women and by 1% among HIV positive women. On the other hand, attaining highest level of education increased the probability of taking up professional care by 1% among both cases; whereas residing in urban areas increased the probability of utilizing professional care by 8% among HIV negative women and by less than 1% among HIV positive women.

The trend was similar during postnatal period in which being richest increased the probability of utilizing professional care by around 1% among both HIV positive and HIV negative women. Attaining highest level of education also increased the probability of taking up professional postnatal care by around 1% among both HIV positive and HIV negative women. Lastly, residing in urban areas increased utilization of professional postnatal care by 4% among HIV negative women and 6% among HIV positive women.

As explained in the methods section, we used Propensity Score Matching (PSM) technique to estimate the effect of HIV positive status on maternal health seeking behavior during antenatal, at birth and postnatal periods. From Table 4 we can see the effect of HIV status on maternal health seeking behavior during the three stages.

**Table 4:** Average Treatment effect on the Treated (ATT) Results

	Professional-care Usage rate for the treated	Professional-care Usage rate for the untreated	ATT: Percentage point (p.p) increase in professional care usage
Antenatal	0.780	0.770	0.0097***
At birth	0.809	0.801	0.0076***
Postnatal	0.771	0.761	0.0099***

***p < 0.01, **p < 0.05, *p < 0.10

The results in Table 4 indicate that being HIV positive increases the probability of utilizing skilled antenatal care by 0.97 percentage points (p.p), representing a professional-care usage rate of about 78%. Being HIV positive increases the probability of utilizing skilled maternal care during birth by 0.76p.p representing a professional-care usage rate of 80.9%. Being HIV positive increases the probability of uptake of skilled postnatal care by 0.9p.p representing a professional-care usage rate of 77.1%. All of these effects are statistically significant.

We used Kernel Matching and Stratification Matching techniques to check for the robustness of the ATT and both techniques produced similar results (see Table 5). A check on the effect of unobservables using the Altonji test also showed that our results were valid (see Table 6).

**Table 5:** Checking for Robustness

	Professional-care Usage rate for the untreated	Professional-care Usage rate for the untreated	**ATT**
ATT using Stratification method			
a) Antenatal	0.867	0.770	0.097***
b) At Birth	0.863	0.801	0.062***
c) Postnatal	0.857	0.761	0.096 **
ATT using Kernel method			
a) Antenatal	0.787	0.770	0.017**
b) At Birth	0.855	0.801	0.054***
c) Postnatal	0.832	0.761	0.071*

***p < 0.01, **p < 0.05, *p < 0.10

**Table 6:** Atonji test. Summary of *P*-Values from different r-levels

Correlation level (r)	Antenatal	Birth	Postnatal
	*P*-Value	*P*-Value	*P*-Value
0.05	0.00	0.00	0.00
0.10	0.00	0.00	0.01
0.15	0.04	0.08	0.07
0.20	0.07	0.08	0.09
0.25	0.31	0.15	0.28

Decision key: To check for significance: see *P*-Value

By focusing on correlations between error terms, we can see the effect that unobservable characteristics could have on treatment and outcome (In this case being HIV positive and taking up professional care respectively). Our *P*-Value indicate that even when we assume a relatively high selection on the basis of unobservables (*r* = 0.15 in this case), the effect of HIV positive status on utilizing professional care remains highly significant. This confirms that our results are not affected by unobservables and thus they are robust

## Discussion

Our aim in this study was to investigate and compare factors associated with the choice of care during antenatal, delivery and postnatal periods (between HIV positive and HIV negative women). We also investigated the effect of HIV positive status on utilization of professional maternal care during antenatal, delivery and postnatal periods. We discuss these results in that order.

Our findings highlight the fact that during antenatal care, at birth and postnatal periods, factors that influence uptake of professional care are similar among HIV negative and HIV positive women. They range from wealth status, level of education and area of residence. In conformity with other studies [26, 27], our findings suggest that regardless of women’s HIV status, being wealth, highly educated, and residing in urban areas increase the probability of utilizing professional care [28]. This suggests as other scholars have argued elsewhere that being more educated and wealthier allows women to overcome barriers of costs which characterize health services in most parts of Africa [29, 30]. This study also agrees with other studies that have shown that in Zambia, women choose to utilize health professionals based on the availability and accessibility of professional health services. Thus in urban areas where health centers are more accessible, women tend to utilize professional health services more compared to rural areas where TBAs are a more readily available [12]. Failure to overcome such structural barriers to care means that some women living with HIV in Zambia fail to access the technically-challenging yet much-needed cesarean procedures during delivery and ART to prevent HIV transmission from mother to child [7, 9]. Further, other scholars including the WHO recommend that professional postnatal care should be adhered to strictly in order to promote the health of HIV positive mothers and their children. It is required that “all infants born to HIV-positive mothers should immediately receive a course of antiretroviral treatment” [9, 31]. Further, depending on the situation, breastfeeding infants must receive a daily dosage of nevirapine and zidovudine from birth for 6 weeks. All children born to HIV positive mothers must also undergo regular HIV tests at 6 weeks, 18 months and one final one after 24 months [10, 31]. Thus given the situation in which some of rural women in Zambia utilize TBAs instead of professional health workers, questions have been raised regarding the practicality of ensuring that the above steps are adhered to strictly [2, 31].

Our results further indicate that being HIV positive increases the probability of uptake of professional care during antenatal, delivery and postnatal periods by a small margin. It is however worth-noting that although the probability of accessing skilled maternal care (as opposed to TBAs) among HIV positive women is higher than those who are HIV negative, the difference is small [4]. It is expected that all women who are HIV positive utilize skilled maternal health services in order to reduce the possibility of optimistic infections, mortality, and to promote PMTCT ([3], p. 13). This is usually through uptake of antiretroviral treatment and caesarean births. Contrary to expectations, our results indicate that there is no substantial difference in maternal-health- seeking behavior between HIV positive and HIV negative women. HIV positive women do not substantially seek professional care more than HIV negative women; this is despite the fact that HIV positive women are more vulnerable than HIV negative women.

Viewed from an ideal perspective, it would seem legitimate to suggest, like many other scholars do, that HIV positive women should always utilize health professionals [3, 31]. However, this will be ignoring the many barriers that HIV positive women are directly faced with in Zambia (low income, inaccessibility of facilities, lack of drugs and personnel in facilities etc.) [32, 33]. In a sense, just as other studies have shown [32], it is counterproductive to encourage and send women to ‘health facilities’ if these facilities lack proper medication, equipment and personnel. It has been documented by previous studies that in Zambia, especially in rural areas, health facilities luck adequate trained personnel, drugs, and equipment [32]. Thus sending women to health facilities which are anything but, is in contradiction with the aims of maternal health promotion programs. In the Zambian case, the focus should be less about ‘institutional care’ but more about ensuring quality and access to feasible and practical care.

### Limitations

One of the limitations of this study is the fact that the HIV status of some women who did not consent to having their blood tested for HIV was unclear and therefore we had to exclude these women from our sample. This means that the final sample was reduced in size and this may have affected the representativeness of the sample. Further, like in most studies that rely on household surveys, we acknowledge that the quality of the data used in this study depends on the women’s ability to correctly recall events during maternity. This means that there is a possibility of recall bias. That notwithstanding, we believe this chapter provides useful insights and grounds for a more careful evaluation of factors associate with choice of antenatal, delivery and postnatal health service between HIV positive and HIV negative women.

## Conclusion

There are no differences between HIV positive and HIV negative women in factors that are associated with choice of care during antenatal, at birth and postnatal periods. Wealth, high level of education and urban-residence seem to increase probability of utilizing professional care among both HIV positive and HIV negative women. Further, we showed that although the probability of utilizing professional care for HIV positive appears to be slightly higher than those who are HIV negative, the difference is almost negligible. This suggests that there is still a large number of HIV positive women who do not have access to PMTCT services. That notwithstanding, we also argue that although institutional care is desirable and an ideal solution for HIV positive women, insisting on exclusive institutional care (which is ideal) while ignoring the many challenges and barriers that exist within professional care in Zambia is counterproductive.

## Abbreviations

AIDS: Acquired Immune Deficiency Syndrome
ART: Antiretroviral Treatment
HIV: Human Immunodeficiency Virus
PMTCT: Prevention of Mother to Child Transmission
SSA: Sub-Saharan Africa
TBA: Traditional Birth Attendants
WHO: World health Organization
